# Finding a Balance Between Being Professional and Being Personal. Experiences of Seven Psychotherapists

**DOI:** 10.17505/jpor.2025.27723

**Published:** 2025-04-01

**Authors:** Mikael Hörnebrant, Åsa Jeppsson, Mats Hilte

**Affiliations:** 1 Department of Social Work, Lund University, Lund, Sweden

**Keywords:** psychotherapist, profession, professional judgement, person, habitus, phenomenology

## Abstract

The aim of this study was to explore factors psychotherapists believe influence their choice of theory, method, and techniques when doing therapeutic work. Semi-structured interviews were conducted with six certified psychotherapists and one psychotherapist in training. The interviews were analyzed using Interpretative Phenomenological Analysis (IPA), a qualitative methodology which explores how individuals make sense of their experiences. With the help of that methodology, we identified four main themes: Congruence, Responsibility, Context, and Flexibility. Our results indicated that practical and theoretical knowledge needs to be synchronized with the individual practitioner’s basic personality. This enables the psychotherapists to experience themselves as professionals, as well as being their authentic self. When the professional and personal identity is joined together, a space is created for greater flexibility in the use of methods and techniques. The study also indicated the existence of a habitus among the psychotherapists embodied in a common language, derived from professional training, supervision and collegial discussions. There was also a consensus among the psychotherapists that they, as professionals, need to be independent and autonomous when doing therapeutic work. Permissive and supportive organizations were seen as an important asset, as well as access to colleagues and networks. The participants in the present study characterized their attitudes to clinical work using keywords such as curiosity, presence, and co-creation. It is suggested that therapists constantly walk a fine line between being an expert and at the same time bracketing their professional knowledge to meet the needs of their clients. A main limitation of the present study was that almost all participants belonged to one therapeutic orientation within the field of psychotherapy (family therapy).

## Introduction

The purpose of this study was to explore which factors psychotherapists believe influence their choice of theory, method, and techniques when doing therapeutic work. Our focus was not the choices made by the individual therapist but rather on the process of choosing, and which factors influenced their choice. We were interested in finding out how they experienced the importance of (a) their professional affiliation, (b) the work context, (c) their own subjectivity and personality, (d) the client’s needs and (e) the context of their choice of theory, method, and techniques. The idea of a relationship and interaction between persons and their environment has been an important notion guiding the study, as well as theories providing us with a contextual understanding of psychotherapeutic work. Central concepts in the study are profession, professional judgement, and habitus.

### Professionalism

Psychotherapist is a protected professional title in Sweden since 1985 and only those who have a psychotherapist degree with subsequent certification from the National Board of Health and Welfare can be employed as a psychotherapist. In common with other professions, it is based on academic education and science, where legitimacy is an important condition for developing trust (Brante, 2011). Trust is built using strict professional integrity, ethical rules, collegiality, and an emphasis on the importance of altruism. Overall, this gives the profession a specific authority, a form of power, and autonomy in making decisions on internal questions.

As described by Evetts (2006), a transformation is taking place, where an older *occupational* professionalism is being replaced by an *organizational* one. According to her, these are two different forms of professionalism, which seem to be competing with one another in the modern world, especially in areas such as health and education where systems of new public management are becoming common (p. 141). In occupational professionalism, the discourse emanates from within the occupational group, where collegial decision-making and self-control is of greatest importance. But in organizational professionalism, the professional’s autonomy and control is challenged by routines, standardized procedures, and a hierarchical structure of decision-making.

Bergmark and Lundström (2011) similarly argue for making a distinction between an illuminated practical-occupation professionalism and a manual-based practical-organizations professionalism. Svensson (2010) underlines that the ability to stand up against bureaucracy and managerial control is an opportunity for experts, with their unique and enduring knowledge.

### Professional Judgement

Håkansson (2015, 2020) contends that we are living in a time of strong belief in the importance of professionalism and measurement, and that we occasionally forget how important personal meetings and emotions can be for professional commitment and action.

A meeting involves those who are affected. A meeting touches. A therapist’s thoughts and feelings play a role, as does his or her attitude. One’s own life experiences leave their mark and influence what the therapist thinks he or she sees and hears. One's own experiences, as well as theoretical affiliation, influence action and meaning-making. Meetings between people bring memories to life. (Håkansson, 2020, p. 5; authors’ translation)

According to Håkansson (2020), professionals can be conceived in two different ways: as subjective beings or as neutral actors unaffected by concrete work experiences. The latter attitude is the most widespread and is likely to influence the treatment practice, as well as the portrayal of actors in the professional context.

Aponte and Kissil (2017) argue that when therapists meet with their clients, they apply both their personal and professional self. This highlights the importance of both being able to identify with and distancing oneself from the client. Tudor-Sandahl (1990) similarly emphasizes the importance of therapists conveying authenticity and honesty, and that the professional and personal self needs to be congruent.

Bornemark (2020) contends that sometimes therapists can get caught up in older themes and topics and lose sight of new storylines. It is a personal challenge to be sensitive to new developments in the client’s story and to sort out what is important and what is not. Professional experience is built upon many layers of experiences of concrete situations, which are embodied in the therapist’s way of thinking and acting. Bornemark (2020) describes judgement as a capacity where professionals are acting in relation to horizons of knowing and not-knowing, to get a glimpse of what is important and being open to actions in new situations.

### Habitus

The sociologist Bourdieu has criticized the use of the term “profession” in scientific research and has argued that we should “*replace* this concept with that of *field*” (Bourdieu & Wacquant, 1992, p. 242). This would mean that, instead of a profession of psychotherapists we should speak about *the field of psychotherapy*. Bourdieu’s theoretical framework also involves another equally basic concept: *habitus*, which is defined as “a system of lasting and transposable dispositions which, integrating past experiences, functions at every moment as a matrix of perceptions, appreciations and actions and makes possible the achievement of infinitely diversified tasks” (Bourdieu & Wacquant, 1992, p. 18). As individuals experience various social milieus, and various forms of professional training, they develop “layers” of habitus or dispositions (Bourdieu & Wacquant, 1992, p. 135) that will guide their practice.

Using this theoretical approach, a sociological analysis of the field of psychotherapy would focus on its various groups of therapists (e.g., different orientations) and clients (e.g., patient organizations) with their respective habitus, as well as governmental authorities in the field (e.g., the National Board of Health and Welfare, and the National Agency for Higher Education), and their various positions, conflicts and alliances. Although such an analysis lies way beyond the aims of the present study, the concept of *habitus* is clearly of relevance for the present study, as it should be expected to find expression when psychotherapists speak about their experiences of what guides them in their clinical work.

### The Present Study

The aim of the present study was to explore what factors psychotherapists believe influence their choice of theory, method, and techniques when doing therapeutic work. Our study incorporated questions such as: (1) Why do therapists end up doing therapy in a certain way? (2) Are they too emotionally involved with their favorite theories and methods?

(3) Do they have the courage to challenge themselves and infuse new theories and methods into their therapeutic work?

## Methods

Our study took place in a field of psychotherapy that we are familiar with and are also participating in. This calls for discretion and critical self-reflection when it comes to interpretations, preconceived notions and values, to maintain a sensitivity to the studied phenomena. In accordance with Interpretive Phenomenological Analysis (Smith et al, 2019), we started from the basic assumption that we live in a world of experiences, and that we (including our informants) gain knowledge through active interpretations of our experiences.

## Participants

We conducted interviews with seven social workers. Six of them were licensed psychotherapists, while the seventh one was undergoing training as a psychotherapist. One of the informants had a psychodynamic orientation, while the rest had a family therapeutic orientation. The informants were recruited among students of a psychotherapist program at Lund University. Table 1 gives an overview of the informants’ work context. The study was conducted by two interviewers.

**Table 1:** Informants’ work context, number of years as practicing psychotherapist, and main target group of clients.

Work context	Number of informants	Number of years as licensed psychotherapist	Main Target Group
Only private practice	2	9 and 9	Individuals and couples
Private employment,	2	3 and 6	Individuals and families
including private practice	2		Individuals and couples
Municipal employment,	3	0, 6 and 6	Families
including private practice	1		Individuals and couples

### Sampling

Strategic, non-random sampling was used. The original criterion of inclusion was that the informants should be licensed psychotherapists with considerable experiences from therapeutic work. We preferred participants from different work settings and with diverse types of employment and employers. Originally, we were satisfied with doing six interviews, but after doing a pilot interview with a psychotherapy student we decided to also include that interview in our empirical data.

## Design and Procedure

The study design was explorative (Kvale & Brinkmann, 2015). We (two interviewers) used semi-structured interviews based on an interview guide with different follow-up questions. The interviews were conducted in February-March 2024 and lasted 55-75 minutes. Interviewer 1 conducted four interviews, and interviewer 2 three interviews. All the interviews were done face-to-face, except the pilot interview, which was a video interview. The interviews were documented with a tape recorder and transcribed in their entirety.

## Ethics

Prior to the interview we send out an information letter to the potential participants. The participants were informed that their participation was voluntary and that they could cancel their participation at any time. All participants provided written consent to participate in the interview study.

Their identities were kept confidential and interview quotes and material have been anonymized.

## Analysis of the Data

This case study aimed to interpret and make sense of psychotherapists’ unique experiences using the qualitative research methodology Interpretative phenomenological analysis (IPA) (Smith et al., 2019). IPA is an inductive research method which starts from concrete examples to develop broader theories. The collected interview data were analyzed to uncover the construction of meaning in a specific context. The analysis was carried through in five steps (Smith et al., 2019).

1. The first step consisted of a review of the transcribed interviews with a goal to understand each individual informant and to interpret their lifeworld. The interviews were read once again in another round from an outsider perspective, with the goal of discovering new themes. This procedure was repeated for all seven transcribed interviews; both interviewers read all seven transcripts.
2. In the next step the two interviewers met and shared with each other the themes they had discovered in the transcripts and then compared the themes between the different informants.
3. Then we entered a more interpretive phase focusing on what meaning the different phenomena had to the informants, but also how our subjective knowledge and experience could affect our interpretations. Patterns and nuances were identified, with a focus on both similarities and differences.
4. The purpose of the next step was to develop an analytic model, where the different themes were related to each other. They were organized into different groups: a structure that consisted of four main themes emerged together with eight associated sub-themes.
5. The last step consisted in showcasing the themes, illustrating them with detailed examples followed by our interpretations, together with reflections on how our perceptions and perspectives could affect the interpretations.

## Results

Table 2 shows the four main themes, congruence, responsibility, context, and flexibility, and their sub-themes.

**Table 2:** Main themes and sub-themes

Main theme	Sub-theme
Congruence	Professional role and identity
	Therapist and method
Responsibility	Responsibility in the therapy room
	Responsibility outside the therapy room
Context	The therapist’s work context
	The therapist’s understanding of the client’s context
Flexibility	The therapist’s flexibility in relation to themselves
	The therapist’s flexibility in relation to the client

To protect the anonymity of our informants, they were provided with new names: Siv, Pia, Liv, Gun, Anna, Lena, and Lars. Our ambition was to be true to the quotations original content, showcasing the informant´s expressed intention. In some of the quotes we have removed specific words and expressions to avoid recognition.

## Congruence

The informants underlined the importance of practical and theoretical knowledge being integrated with the individual therapist’s basic personality, allowing them to be personal and professional at the same time. They also agreed that the psychotherapist role is characterized by qualities such as being curious, exploratory, and committed.

### Professional role and identity

Several of the informants used their primary education as a framework to describe their professional identity, either as a social worker, family therapist, systemic psychotherapist, or as a psychodynamic therapist. The interpretation of their role as a professional therapist was something that, besides theoretical knowledge, needed time and experience to mature.

My role as a psychotherapist is something that has developed over the years through experience and the support of theoretical and practical knowledge. Greater confidence has appeared, and I feel calmer. (Gun)

An impression emerged that the therapists brought their personal and basic values into the therapeutic relationship.

I think that it’s like you have a political affiliation or that you have a basis that these are my values, you cannot just replace them. I couldn’t do anything that didn’t feel right to me, because then I wouldn’t be good and it wouldn’t have worked. (Siv)

Many of the informants shared the opinion that there had to be a consistency between the psychotherapist´s experience of their professional role and their subjective way of doing therapeutic work.

Personality is especially important. It’s important in building trust, for people to feel safe with who I am. I try to be quite open, sharing myself without becoming too private. (Liv)

With an increased confidence in professional methods and techniques, accomplished through regular knowledge acquisition and recurring opportunities for reflection, conditions were generated for being open and perceptive in relation to the client. At the same time, informants pointed out the importance of maintaining boundaries between their professional and private self. It was important to be personal and authentic in relation to the client, but also to avoid sharing their private self.

I am very personal but never private in my meeting with the client and I know exactly where that line is. (Pia)

### Therapist and method

All the informants had a theoretical platform or basic work model guiding their therapeutic work. Five of the informants admitted that their choice of therapeutic model was quite coincidental and came early in their treatment careers.

The method helps me uphold a common thread and I don´t lose interest. Curiosity and interest are incredibly important. When I notice that I am zooming out from a conversation, and there can be various reasons for that, the method helps finding my way back. (Anna)

Lena underlined the importance of using methods in therapeutic work:

I use methods to understand, they are stored within me. If that wasn´t the case, I think you would lose it, if things happen to you and you don´t try to understand. (Lena)

Over the years, the preferred methods have been personalized as their own and formulated in their own words. Consequently, the methods and their personal and professional style are experienced as intertwined.

My experience from when I had worked for a shorter time and had a method, I applied it to many families who met the criteria. I can meet similar families today and choose to work using a different method. Because now I have more options to choose from. (Liv)

When the professional therapist has become familiar with certain therapeutic tools, i.e. theories, methods and techniques, new tools are made available, shedding light on the older ones.

Our work is manual-based, but within this framework there is great freedom. If you work systematically and with families, you can work quite freely. (Lars)

When therapists were confident using their therapeutic models, their courage increased to remove aspects that didn’t work from their professional toolbox. To have professional freedom and the space to act was emphasized by all informants as important in their work.

## Responsibility

All informants agreed that the concept of responsibility was of crucial importance in their work and in their role as a psychotherapist. It was important to take responsibility for creating a trusting relationship in order for clients to feel secure.

### Responsibility in the therapy room

The informants concurred that the therapists were responsible for leading and managing the therapeutic work, helping clients to address relevant topics in a way that was helpful to them.

I set up clear frameworks for the conversation and help the client formulate what is important to them. (Siv)

I contribute by helping them devote themselves to the right things, to steer the conversation, to listen with a trained ear to what we need to focus on. Yes, to pilot and steer as well, so it is effective in the end. (Anna)

The informants also highlighted the importance of: (1) being aware of themselves in relation to the client, (2) being responsible and (3) grounding their actions on both their professional and private self.

I have a responsibility in the room and need to feel secure in which direction we should go. I also have a responsibility to deal emotionally with what the client brings. (Lars)

### Responsibility outside the therapy room

The informants reported they were responsible for being up to date when it comes to professional training and discussions of methods, in order to be well prepared for practicing good therapy.

I am continuing my education to better meet the needs of clients and to have more tools in my toolbox. I need to be aware of what is happening. (Liv)

Informants highlighted that as therapists they have a responsibility to keep the client’s perspective alive in various contexts.

When the client comes from tangled and difficult circumstances, it is important to stay connected even between sessions in order not to lose sight of the client. (Lars)

I sometimes help patients to another healthcare provider when necessary. It is a part of my professional responsibility being able to guide them. (Lena)

Several of the informants mentioned how important it was to disseminate knowledge within organizations, to managers and politicians, but also in relation to other community efforts and to convey the importance of competence development among psychotherapists. As one informant put it:

It is also important to preserve the therapeutic space for action in various contexts bearing in mind the increase of manualized methods within the social services. (Siv)

## Context

There was a mutual understanding among the informants that the context in which they work and who they meet with is important in therapeutic work.

### The therapist’s work context

The informants worked in different organizations under varying working conditions, either as full or partial employees or as self-employed to varying degrees (Table 1). The availability of colleagues and networks for discussions and reflections on professional matters was perceived as a key factor for professional development and job satisfaction. The freedom of therapeutic action included the possibility, not only to choose therapeutic tools, but also freedom of action in terms of the number of sessions and amount of time devoted to therapeutic work.

Previously, there was no limit in number of sessions, and we were quite free to use different toolboxes, but it is different now, we have much less methodological support. I need to experience some sense of freedom within the organization to be able to work in a way I am comfortable with, otherwise it won’t turn out well. It is a kind of disorder in the organization when certain methods become fashionable and everyone must comply. This can change suddenly and the fashionable method is replaced with a new one. When reorganizations are made and new directives are issued from above, it does not always turn out well for the people we are supposed to help. (Anna)

Five of the informants had experiences from working in their own private practice. They described the difference between private and municipal work in terms of getting rid of unwanted tasks, external control, prescribed therapeutic methods and unannounced reorganizations.

When I worked in psychiatry and they started with reorganizations, then it didn’t feel satisfactory anymore, so I started my own private practice instead. A couple of us work in the same agency, are a bit like a hairdressing salon, have different therapeutic orientations. We give each other advice and as a psychodynamic therapist I can, for example, introduce CBT elements into the treatment. It is important for me to have colleagues and being in a context where you can build a network. (Lena)

The organization must be supportive on different levels and the support must also be anchored higher up in the decision-making hierarchy.

At my workplace, we have the goal to maintain a prominent level of competence, giving the staff the opportunity to develop their skills, which is a management decision anchored higher up in the organization. (Liv)

Having the opportunity to decide over one’s own professional work and being part of a working community built upon trust and support, appeared to be of special importance.

### The therapist’s understanding of the client’s context.

According to the informants, the client´s resources regarding education, finances and motivation were important factors in therapeutic work. Besides that, all informants concurred that the way clients were recruited was crucial for how the introduction and treatment was organized.

I always need to consider the context in which we meet. When people turn to us by themselves, it becomes important to have a conversation and questions that allow me to understand what will be important to them. In cases where they show up for treatment and the social worker is worried, I can be more in charge and have an agenda of what we need to talk about. I need to be factual about the assignment and the framework, repeat it so that it becomes understandable. (Siv)

A decisive condition was if the client had made an application on their own or was receiving assistance from the social services, which can be voluntary, but also subordinated to underlying threats of constraint.

The clients don’t always want to be in treatment and show up because the social services think they have a problem. The motivation is not always present, and I must figure out how the clients want to use my services. In my private practice, it is a different target group. They are well educated, have a good economy, and have a different motivation when they show up. (Pia)

Based on the informants’ answers, families did not seek therapy privately, instead they turned to the social services. It was more common for couples and individuals to seek privately practicing therapists.

In my personal practice there are mostly couples, because the families from social services cannot afford a private therapist. The clients in our agency have applied by themselves and are quite clear about their goals. (Lars)

Liv, another informant, made a comparison between her work as an employee and working in her own practice.

When you are an employee there is more time to build a relationship with the client than there is in my own practice. In a private practice, they can terminate the contact whenever they want to. They have slightly different expectations and requirements when they apply themselves, they are clearer about how they want to be helped. (Liv)

## Flexibility

When it comes to meeting the needs of the clients, and the psychotherapist’s own aspirations to provide good therapy, flexibility was a practical tool. This is valid for both the therapist’s flexibility in relation to themselves and the therapist’s flexibility in relation to the client.

### The therapist flexibility in relation to themselves

When the informants described their role as therapists they emphasized a dimension of constant professional development, meaning that they as persons and psychotherapists were exposed to new experiences and insights.

I use my own self in the conversations, I dare to be close, to touch and being touched. It is not possible to be neutral, the feelings are helpful, and they show which way we should go. (Siv)

Being in contact with the client, as well as being emotionally present and guided by one’s own emotions, was highlighted as important in the informants’ stories.

To be in contact with oneself, to be present and curious in the room and to use one’s sense of humor. (Lars)

It was emphasized by the informants that education and continuous replenishment were significant and helped them to maintain interest and energy in their therapeutic work. There was a desire for professional development and learning, not just for their own sake, but also to be more helpful in relations to clients.

Mentoring is important to gain insight into oneself. (Pia)

Judging by the interviews, the therapists are constantly searching for renewal of knowledge and embrace the presumption that therapists will never be full-fledged, because to some extent they are in a state of constant movement and flexibility.

### The therapist flexibility in relation to the client

Overall, according to the informants, the clients’ needs, abilities, and motivations were often different from each other. Therefore, the conditions were always shifting in the therapeutic relationship.

It cannot become a routine so that people feel that I only mirror a manual or program. (Gun)

Informants highlighted that there were families who needed an active and controlling therapist, whilst in other relationships there was a need for co-creation, where the therapist were more focused on keeping the process going.

It is not who I am, I must adapt to the person I meet, I think. I cannot keep the same posture all the time. Sometimes you must be more on and sometimes you must be more off. (Lena)

According to the informants the palette of techniques used by the therapists can be exceptionally large and varied.

I am much more careful today when it comes to asking clients how it turned out for them and if the therapy works. Sometimes I get feedback that makes me think differently, then maybe we will do something different the next time we meet (Liv)

A prevailing attitude among the informants was that therapy must be a co-creation together with the clients, where they have influence over the therapeutic process. Siv summarizes her relationship to the client:

I am not an expert on other people’s lives, but I can use my knowledge to try to be useful, but first I need to understand what is important to you. (Siv)

## Discussion

The aim of the present study was to explore factors which psychotherapists believe influence their choice of theory, method, and techniques in their practical work – or more generally: why they end up doing therapy in a certain way. The analysis of the interviews with the psychotherapists led to the identification of four main themes: Congruence, Responsibility, Context, and Flexibility. In the following discussion, these themes are discussed in terms of the original research questions. First, we discuss how the interaction between the subjective and the professional dimensions impacts the therapist’s flexibility, then how the organization and the social context impact therapists’ room for action, and finally how their professional judgement is adapted to the clients’ situation and needs.

As to the first question, the informants clearly have a basic theoretical platform and method which have stayed with them over time and has become part of them as therapists. The method serves as a guiding principle and helps them structure their therapeutic work and make things understandable. There is a great aspiration for professional development among the informants, to learn more about different methods and being helpful to different clients. But the methods are not of primary importance to therapists in their interaction with clients. The priority is to find a way of being together with the client based on presence, curiosity, openness, helpfulness, and client orientation. This way of being together with the client, creating a special space for action, is shared by most of the therapists and indicates the existence of a therapeutic and professional culture. This culture provides them with considerable flexibility in their choice of method, helping them to adapt to the client’s needs and situation.

Second, the context of the therapeutic meeting, the purpose and framework of the therapeutic work, influence how the therapists take on their professional role and how methods are introduced. There are organizations today that are premised on the idea that the client is the expert on their own life, and it has become increasingly important to create a dialogical meeting with the client. At the same time, there are organizations which provide manual-based treatments for specific problems, where the method is not optional, neither for the therapist nor the client. The informants described the importance of being autonomous in their role as professionals, being able to influence their therapeutic work and the choice of methods. The informants underlined the importance of using their own personal self as a tool and that their personality needs to conform with their therapeutic role. At the same time, they pointed out the importance of boundaries between their private and professional life. According to the therapists the clients often look up to them as experts who will help them solve their problems. The informants also described therapeutic work as walking on a thin line between being an expert and a not-knowing attitude. Being an expert, the therapist leads the conversation, making sure the dialogue is about relevant things, helping clients formulate treatment goals and taking responsibility for being in treatment.

Third, the therapist´s judgement is subjective and affects how they interpret and implement theories and methods in specific situations. However, this doesn’t take place in a social vacuum but interacts with many other contextual factors. In fact, these are mechanisms that can be hard to notice and become aware of. It requires dialogues with colleagues and self-reflection to grasp one’s own judgmental structure. Our study indicates the existence of a habitus among the informants dependent on social networks, discussion with colleagues, a mutual field of practice and similar professional education and traditions. Therapeutic work is also executed against the backdrop of cultural beliefs embedded in the organization. These prescribe what a psychotherapist can and should do, as well as which methods are best suited and how therapists should relate to them.

Figure 1 gives an overview of the factors and processes that, according to our findings, can influence the psychotherapist’s choice of methods, theories, and techniques. It illustrates the different kinds of relationships that therapists construct in relation to the context (occupational or organizational professionalism) as well as in relation to the client (co-creative client-centered or expert/manual-based). The tension between occupational and organizational professionalism was deeply felt by the informants. One of the therapists compared organizational professionalism to “a kind of disorder in the organization when certain methods become fashionable, and everyone must comply”. Some informants also experienced tensions around being an expert or taking a more client-centered approach. And one of them underlined the need to be cautious in that respect: “I am not an expert on other people’s lives, but I can use my knowledge to try to be useful, but first I need to understand what is important to you.”

### Comparison with Previous Studies

The informants underlined the importance of finding a match between the treatment methods and the therapist’s personality. This is in accordance with Simon’s (2006) suggestion that therapists achieve maximum effectiveness if they commit themselves to a treatment model whose underlying worldview closely matches their own personal worldview. In addition, the methods should also be adjusted to the clients, which requires methodological flexibility. When therapists choose a method that is consistent with them as a person (e.g., values and personal style) this is synonymous with therapists’ applying their best professional self. This is consistent with our findings that therapeutic ideas and methods need to have a personal base. This contributes to the development of professional authenticity and the building of trust and security in the role as a psychotherapist.

Blow et al. (2007) argues that the cooperation between therapists and clients is of primary importance and that the choice of therapeutic method is secondary. When therapists have access to different treatment methods, they have greater opportunities to help clients in their quest for personal change. This is consistent with our informants’ experiences that adjustment and co-creation are important doing therapy and developing an understanding of what’s important in the client’s life. In our study the therapist’s professional and personal self had to be synchronized to radiate authenticity and genuineness in the therapeutic meeting. This is also consistent with Heinonen and Nissen-Lie’s (2020) conclusion, in a systematic review of research on therapist pre-treatment characteristics that predict patient outcomes, that effective therapists show professionally cultivated interpersonal capacities, which are likely rooted in their personal lives and attachment history. Bernhardt et al. (2019) also concluded in their study that personality traits such as seeing oneself as a helper, being a present listener, someone who can manage anger and rejection, and being able to balance distance and closeness, are all decisive in therapeutic work.

The informants describe their professional development as a process that involves gaining new knowledge and new experiences, increasing their professional versatility making them more disposed in helping different clients with various kinds of problem. In many studies (Kumaria et al., 2018; Lorentzen et al., 2011; Rønnestad & Skovhult, 2003) practicing therapy is described as an ongoing acquisition of theoretical knowledge, life experiences and joining with professional networks. Kissil et al. (2018) argues that an increased self-awareness of personal difficulties and vulnerabilities are beneficial conditions for developing greater self-confidence in professional therapeutic work. Gold and Gold (2023) studied therapists’ lack of faith in their competences and concluded that self-doubt can have both positive and negative consequences. These consequences are distributed on a spectrum, where the positive extreme entails being spurred to professional development, while the negative extreme involves harsh self-criticism.

### Limitations

The participants were not a representative sample of psychotherapists, and almost all of them were from one specific orientation (family therapy). We don’t know what the outcome would have been if we had included therapists from other orientations, or if we had used a randomly selected sample of family therapists.

### Conclusion

Our study highlights the emphasis psychotherapists put on the opportunity to synthesize and integrate abstract theories and methods with their own personal experiences and personality. The selection of treatment method is important, but initially it’s not what is most important to the client. Clients need to feel that therapists want to help them with what they experience as problematic. One client said, “I don´t know if you can help me, but if I didn’t believe that you really wanted to help me, I wouldn’t have come back to you.” Treatment methods are important tools in therapeutic work, being part of the psychotherapist’s toolbox, generated by education, practice, and being part of different practice settings. It is an advantage to have access to different therapeutic methods. If you only have a hammer in your toolbox, then you will only see the nails.

More research needs to be conducted on the practice of professional judgement among psychotherapists. A pertinent question is how theoretical models and methods are best integrated with the therapeutic self. In-depth knowledge about these matters could increase our understanding of how therapeutic methods can be adapted to and more helpful to the clients. This requires theories about the interpersonal relationship between psychotherapists and clients, the intrapersonal processes on behalf of the psychotherapists, and the social context in which the therapeutic work is taking place. If organizational professionalism challenges the psychotherapist’s control over their work, this could circumscribe their freedom to decide what methods to apply in their therapeutic work. One consequence of this could be that psychotherapists drop out from municipal organizations, which in turn might lead to a loss of competence and deterioration of quality in therapeutic work. Further research should investigate how municipal organizations can tap into and take advantage of professional therapists’ knowledge and experience.

## Action editor

Lars-Gunnar Lundh served as action editor for this article.

## Open access

This article is distributed under the terms of the Creative Commons Attribution 4.0 International License (http://creativecommons.org/licenses/by/4.0/), which permits unrestricted use, distribution, and reproduction in any medium, provided you give appropriate credit to the original author(s) and the source, provide a link to the Creative Commons license, and indicate if changes were made.
